# Intelligent Design *versus* Evolution

**DOI:** 10.5041/RMMJ.10007

**Published:** 2010-07-02

**Authors:** Nathan Aviezer

**Affiliations:** Department of Physics, Bar-Ilan University, Ramat-Gan, Israel

**Keywords:** intelligent design, evolution, irreducible complexity, science, religion

## Abstract

Intelligent Design (ID) burst onto the scene in 1996, with the publication of *Darwin’s Black Box* by Michael Behe. Since then, there has been a plethora of articles written about ID, both pro and con. However, most of the articles critical of ID deal with peripheral issues, such as whether ID is just another form of creationism or whether ID qualifies as science or whether ID should be taught in public schools. It is our view that the central issue is whether the basic claim of ID is correct. Our goal is fourfold: (I) to show that most of the proposed refutations of ID are unconvincing and/or incorrect, (II) to describe the single fundamental error of ID, (III) to discuss the historic tradition surrounding the ID controversy, showing that ID is an example of a “god-of-the-gaps” argument, and (IV) to place the ID controversy in the larger context of proposed proofs for the existence of God, with the emphasis on Jewish tradition.

## INTRODUCTION

The concept of Intelligent Design (ID) was proposed in 1996 by biochemist Michael Behe in his book, *Darwin’s Black Box, the Biochemical Challenge to Evolution*. Behe claimed to have discovered an *ironclad proof* for the existence of a supernatural being, whom he called the “Intelligent Designer.” His studies of the living cell led Behe to conclude that Darwinian evolution cannot explain many biochemical reactions that take place in the cell; only ID can. Although Behe studiously refrained from identifying the Intelligent Designer, the widespread understanding is that the Intelligent Designer is God.

Behe’s proposed proof that the cell could not have formed through Darwinian evolution, generated enormous interest (reported in *Newsweek*, *U.S. News & World Report*, *New York Times*, *Commentary*, *National Review* and many other periodicals).

Michael Behe is a creationist’s dream come true. Unlike previous religious “scientists” who attacked evolution, Behe is a Professor of Biochemistry at a respected university, a research scientist who does experiments, is awarded grants and publishes papers in international science journals. Moreover, his book is extremely well written, cleverly argued, and shows his obvious expertise in biochemistry. Indeed, Behe’s book is the most sophisticated attack on evolution to appear in recent years. It has revived the hopes of the creationists – here is a professional biochemist claiming that the Darwinists are all wrong about evolution.

The present article focuses on various aspects of Intelligent Design. What exactly has Behe claimed and why is this claim wrong? What is the history of ID and what can we learn from this history? What did the critics say and what should they have said? What important implications would follow if ID were indeed correct?

## IMPORTANT AND UNIMPORTANT ISSUES

Some issues that are irrelevant to Behe’s claim have, unfortunately, occupied the attention of many of those involved in the ID debate. It does not matter whether ID is or is not science; it does not matter whether ID is or is not creationism; it does not matter whether or not ID should be taught in the public schools. The only question that is important is *whether or not the claim of ID is correct*.

The scientific world was immediately up in arms against Behe’s book. He was ridiculed for claiming^1^ that his discovery is “so significant that it must be ranked as one of the greatest achievements in the history of science”, rivaling “those of Newton, Einstein, Lavoisier, Schroedinger, and Pasteur.” Many scientists wrote that one should dismiss out of hand the claim of ID because Behe invoked a supernatural being to explain an important part of the physical world.

Much less effort was spent in examining whether Behe’s claim is correct. For example, philosopher of science Michael Ruse^2^ recently published an essay discussing ID. His opening sentence is the following: “We need to answer two questions: What is ID, and is it science?” However, I believe that what we really need to answer is whether the claim of ID is correct.

If ID were correct, then Behe would be perfectly justified in asserting that ID is the greatest challenge imaginable, and not just to evolution, but to science itself. ID would show that the central assumption of science for hundreds of years was wrong! Since the time of Newton, the enterprise of science has been based on the assumption that the laws of nature are sufficient to explain all physical phenomena, without the need to invoke supernatural beings. If this assumption were proven to be incorrect, this would indeed be “one of the greatest achievements in the history of science,” rivaling “the achievements of Newton, Einstein,” and the others. Behe did not exaggerate in the slightest regarding the significance of his claim. Therefore, it is of utmost importance to establish whether or not the claim of ID is correct.

## NAME-CALLING

One of the most unfortunate features of the widespread criticism of ID is the persistent name-calling to which ID has been subjected. ID has repeatedly been called a creationist idea. The purpose of this terminology is clear. The creationists refuse to accept even well-established science if it contradicts their understanding of the literal meaning of the words of Genesis. Therefore, referring to ID as a creationist doctrine immediately labels ID as standing in opposition to science. By this name-calling device, the critics of ID have already won the battle in the minds of the public without having to deal with the real issue of whether or not the claim of ID is correct.

For example, philosopher and historian of science Robert Pennock edited a volume about ID, entitled “*Intelligent Design Creationism and Its Critics*.” The very title of the book characterizes ID as a type of creationism. The expression “intelligent design creationism” is repeated so often that it merited an acronym (IDC). Pennock^3^ describes ID as follows: “The last decade of the millennium saw the arrival of a new player in the creation/evolution debate – the intelligent design movement.”

The essays in Pennock’s book continue this sorry tradition. In her very first paragraph, philosopher Barbara Forrest^4^ informs the reader that: “Intelligent design theory is the most recent – and most dangerous – manifestation of creationism.”

One wonders just what could be “dangerous” about the ID claim regarding the origin of the living cell. It is quite ironic that the same charge – *dangerous* – that is here being hurled against ID, has also been used by creationists against evolution. Creationists point out that the Nazis used the Darwinian concept of “survival of the fittest” to justify their mass murder of millions of “less fit” people, including Jews, gypsies, and Slavs. Therefore, creationists claim, accepting Darwinism is *dangerous* because it can lead to Nazism. And now we are told that also ID is *dangerous*!

Probably the most blatant example of name-calling in this volume is the essay by philosopher Philip Kitcher,^5^ bearing the sarcastic title “Born-Again Creationism.” This essay is literally riddled with snide, derogatory remarks and with errors in his calculation of probabilities, but that is not my concern here.

Sometimes a different type of name-calling is used. Behe is also accused of invoking the “argument from design,” a thousand-year-old “proof” for the existence of God that was refuted long ago. For example, evolutionary biologist Kenneth Miller^6^ starts his discussion of Behe’s book as follows: “The heart and soul of Behe’s treatise against evolution is neither new nor novel. It is the ‘argument from design,’ the oldest and best rhetorical weapon against evolution… Behe has dusted off the argument from design, spiffed it up with the terminology of modern biochemistry, and then applied it to the proteins and macromolecular machines that run the living cell.”

What is the “argument from design”? First, note that the “argument from design” has no connection whatsoever with “Intelligent Design,” except for sharing the word “design” in their title. Also, note that the word “argument” does not denote disagreement; it is an old English word for “proof.” The “argument from design” is a proposed proof for the existence of God based on the complexity of the world. The argument claims that complex structures that carry out specialized tasks never form all by themselves; they always have a maker. Consider a watch, wrote British theologian William Paley in 1803. In the same way that a watch proves the existence of a watchmaker, so goes the argument, the extreme complexity of the universe proves the existence of its Maker.

We now know that this “proof” is wrong. In all fields of science, we observe extremely complex structures that carry out specialized tasks (complex molecules, intricate crystals, vertex structure of type II superconductors, fractal symmetry, etc.) *that form all by themselves*, given the raw materials and suitable temperatures. Therefore, it is sufficient for Miller to assert that Behe bases his claim on the argument from design, and the reader is already convinced that Behe is wrong.

Anyone whose knowledge of Behe’s thesis comes from Miller’s book, would be quite astonished to learn that Behe *explicitly rejects* the argument from design. Behe emphasizes that it is *not complexity* that is the basis for his claims about ID. Rather, it is a *particular type* of complexity which he calls “irreducible complexity.” Behe categorically agrees that extremely complex structures can evolve gradually according to the standard Darwinian mechanism for evolution, but not when *irreducible* complexity is involved. Moreover, a system can be quite simple in the sense implied by the argument from design, and still be irreducibly complex in the sense that Behe means.

## IRREDUCIBLE COMPLEXITY

When Behe speaks of irreducible complexity (IC), what does he mean? How does IC differ from the usual forms of complexity? What is the basis for his claim that IC *cannot be explained* by the standard Darwinian evolutionary theory and that only ID can account for the IC that is found in the living cell?

Darwinian evolution works by the chance appearance of a favorable mutation in the genetic makeup of an animal. The favorable mutation enhances the animal’s chances for survival by making the animal a bit stronger, faster, or less susceptible to disease, etc. Therefore, the animal with the favorable mutation will probably live to reproduce the next generation, and this mutation will become incorporated into the species gene pool. The accumulation of many favorable mutations over many generations brings about large changes in the animal, eventually leading to an entirely new species.

The key point is that according to Darwinism, *only those mutations that enhance the animal’s chances for survival* become incorporated into the gene pool. It is unlikely that a mutation that provides no survival advantage will be passed on to the next generation.

Behe asserts that the gradual accumulation of favorable mutations *cannot* explain the development of many vital biochemical mechanisms. Among the various examples cited by Behe is the mechanism for blood clotting. A large number of chemical reactions are involved in blood clotting, and – here is the crucial point – *if even one of these reactions does not occur*, the blood will not clot. Therefore, claims Behe, the mechanism for blood clotting could not have evolved gradually through a series of mutations, *with each mutation providing an additional survival advantage to the animal*. Each such mutation would, by itself, be useless. *All the mutations* have to be present to be of any use to the animal because *every one* of the reactions involved in blood clotting must occur or the blood will not clot.

The mechanism for blood clotting is called “irreducible” because it *cannot be reduced to a series of steps* with each step affording an additional survival advantage. Rather, the complete blood-clotting mechanism had to appear in the species gene pool *all at once*. According to Behe, this implies design – “Intelligent Design.”

It is important to note that even a relatively simple system, *consisting of only two parts*, can be an irreducibly complex system, if *both parts are necessary* for the system to function. Behe discusses the mousetrap as a classic example of an IC system. There is clearly nothing very complex about a mousetrap. This example serves to confirm that Behe’s assertion that ID *has nothing at all to do with the argument from design*.

## UNCONVINCING REFUTATIONS OF ID

Some of the proposed refutations of ID are rather unconvincing. Consider the following refutation (which has many adherents, just look in Google), proposed by biologist Robert Dorit^7^:

“Many of the proteins of the eye lens, for example, began their careers doing something completely different and unrelated to vision. Evolution is a creative scavenger, taking what is available and putting it to new use. The correct metaphor for the Darwinian process is not that of a First World engineer, but that of a Third World auto mechanic who will get your car running again, but *only if the parts already lying around can be used for the repair*” (emphasis added).

There is a very important implication in the italicized words. What if the necessary parts were *not* already lying around? Dorit’s argument implies that it would then be *impossible* to produce the corresponding IC system by Darwinian evolution. This would be an enormous limitation to the evolutionary process.

Evolutionary biologist H. Allen Orr^8^ dismisses the above proposed refutation of ID: “We might think that some of the parts of an irreducibly complex system evolved step by step for some other purpose and were then recruited wholesale to a new function [which is precisely what Dorit proposed]. But this is unlikely. You may as well hope that half your car’s transmission will suddenly help out in the airbag department. Such things might happen very, very rarely, but they surely do not offer a general solution to irreducible complexity.”

## ORR’S REFUTATION OF ID

Orr then shows how an IC system can indeed evolve through a gradual Darwinian process, *without having to assume* that the “necessary parts were already lying about,” ready to be scavenged to fabricate the IC system. That is, an IC system can be built up *gradually* by adding parts in a way that each part offers an additional advantage, *even though the final system is IC.*

Consider an IC system consisting of several parts, and assume that each part is produced through a genetic mutation. Although this is a simplification of how genes work, this description is quite sufficient for our purposes.

In the distant past, the system may have consisted of only one part, say part A. The system worked, although not too well. A genetic mutation then produced part B, which led to a somewhat improved system, consisting of A plus B. This improved system is *not* IC, because it will function even without part B. A second genetic mutation then transformed A into A*, which led to a further small improvement of the system. However – *and this is the crucial point* – A* will not work unless B is present. Therefore, the present system, consisting of A* plus B, *is* IC because both A* and B are necessary for the system to function.

We have thus shown how an IC system can be produced by means of gradual evolution, with each mutation leading to a small improvement in the system, although the final system (A* plus B) *will not function at all* unless both its parts are present. Therefore, we are done. The claim of ID – *that this is impossible –* has been refuted.

Let’s continue. A third genetic mutation produces part C, which leads to a further small improvement. This system is *not* IC, because it will function even without part C. A fourth mutation then transforms B into B*, yielding yet another small improvement. However, B* will not work unless C is present. Therefore, the improved system (consisting of A* plus B* plus C) *is* IC because *all three parts are necessary for the system to function*. Nevertheless, this IC system was produced *by a series of gradual improvements*, in the best tradition of Darwinian evolution.

This process can be continued to gradually produce a ten-part IC system, consisting of A* plus B* plus C* plus D* plus E* plus F* plus G* plus H* plus I* plus J*. *And there was no need* “to use parts that are already lying around.”

A very important feature of this procedure concerns its *irreversibility*. After the system has been formed, all we see is the final product. We have no way of knowing in what order the ten parts were formed, or what were the intermediate parts (A, B, C, D, E, F, G, H, I and J). Once the scaffolding has been removed, there is no way to determine how the IC building was constructed. But, in contradiction to the claim of ID, its construction was certainly possible!

## TEACHING ID IN THE PUBLIC-SCHOOL SCIENCE CLASSROOM

A much debated question relates to teaching ID in the science classroom. Shouldn’t one teach ID in the public schools because, as former President George W. Bush^9^ enticingly suggested, “an essential part of education is to expose the student to different schools of thought.” Aren’t creationists right when they say that a central feature of a liberal education is to acquaint the student with various points of view?

The flaw in this suggestion is the following. In other disciplines (philosophy, theology, political science, economics, etc.), there exists more than one legitimate school of thought. In science, however, there is *only one correct explanation* for each physical phenomenon.

Phlogiston theory is *not* a “different point of view” to explain the rusting of metals, to which “the student should be exposed to give him a liberal education.” Phlogiston theory is *wrong*! Chemical oxidation is *the only correct explanation* for rusting. Similarly, caloric theory is *wrong*! And the ether theory is *wrong*! Therefore, these incorrect theories *are never taught* in the science classroom, except perhaps to explain to the student *why* these theories are wrong.

It should be noted that Newton’s mechanics is *not wrong*. Rather, Newtonian mechanics is a highly accurate approximation to Einstein’s theory of relativity and to quantum theory (except for extremely high speeds or extremely tiny particles). In fact, Newton’s theory is so accurate over such a wide range of circumstances that every student of physics is required to learn Newtonian mechanics. In complete contrast to this situation, caloric theory, phlogiston theory, and ether theory are *not* approximations to some correct theory. *They are simply wrong*.

## HISTORICAL PRECEDENTS FOR ID

Intelligent Design is not a new concept. Ancient peoples observed phenomena that seemed completely inexplicable to them, and they postulated supernatural beings (analogous to today’s Intelligent Designer) to explain these phenomena. Raging seas, towering waves, daily tides, terrifying hurricanes – all these seemed to have no possible explanation other than the activities of the “god of the seas.” The dazzling sun, whose brilliance provides the light, heat and energy that makes life on earth possible, seemed to have no explanation other than the “sun god.” The list goes on and on, accounting for the vast pantheon of gods that characterized the ancient world.

The ancients asked sophisticated questions about the world in which they lived. If their questions seem primitive today, it is only in the hindsight of modern science. Consider the following example. I am holding a pen. If I let go, the pen will fall to the floor. Already at age four, my grandson knows that if he lets go of his ball, it will fall. Everyone knows that an object falls unless held up by some entity. That’s just common sense.

The ancients asked: Why does the earth itself not fall? They answered that the reason must be because the earth is being held up by some divine entity, a god whom the Greeks named Atlas. Moreover, they understood that one *cannot* ask: Why does Atlas not fall? As a god, Atlas was not bound by the laws of falling; he may remain suspended at will.

## THE SITUATION TODAY

Michael Behe is carrying on this tradition. He could not imagine any possible physical explanation for the IC of the living cell. Therefore, he postulated a supernatural being. Had Behe lived in the ancient world, he might have referred to this supernatural being as the “god of the cell.” However, in the twentieth century, such terminology is unbecoming. Intelligent Designer sounds much better.

One would think that something would have been learned from past experience. It has been shown time and again that physical phenomena that are not understood at the moment *do* become understood subsequently *within the laws of nature*. Science has an excellent track record and is not to be abandoned lightly. If scientists do not understand some particular phenomenon, they think harder. They don’t throw up their hands and give up the search.

In complete contrast to this traditional approach of science, the proponents of ID have abandoned the search for a scientific explanation for IC (that is, within the laws of nature) and have proposed a supernatural explanation instead (that is, ID).

## PROOFS FOR THE EXISTENCE OF GOD

Seeking proofs for the existence of God sounds quaint to the modern ear, but it was a matter of great importance to medieval philosophers, both Jewish (e.g., Maimonides) and Christian (e.g., Thomas Aquinas). Why was it so important to these outstanding thinkers to be able to prove that God exists?

To answer this question, one must return to the period that preceded modern science. In the ancient world, discovering the laws of nature by experimentation was a foreign idea. The mathematicians had discovered the laws of geometry by pure reason, and it was viewed as self-evident that this was the appropriate method for studying the physical universe as well. Indeed, performing careful experiments and carrying out detailed observations seemed unbecoming to the philosopher. His realm of activity was the mind; only a servant or an artisan would “get his hands dirty” with the many menial tasks required to carry out an experiment. An exception was astronomy, where the ancients excelled at observing the motion of the heavenly bodies, the great handiwork of the Creator. Since the heavenly bodies were exalted, observing their motion could not be degrading. However, examining earthly objects was deemed inappropriate for the philosopher – the thinker. Thus, we find in philosophical texts that in contrast to a man, a woman has only twenty teeth (the correct number for both sexes is thirty-two). It did not occur to the scholastic philosopher to count a woman’s teeth. Such a prosaic act was completely unnecessary. Everything could be determined by reason, logic and thought.

The above approach was not limited to the study of the universe. It was believed that *all* fundamental questions could be answered by logical deduction and pure reason. Since medieval theologians believed that God exists, they naturally assumed that His existence must be susceptible to rigorous proof. Indeed, in their eyes, the inability to prove that God exists might even cast doubt on His existence.

Because of their reverent attitude towards the power of logic, many Jewish philosophers devoted considerable effort to arguments intended to prove that God exists. Although this subject is nowhere discussed in the Bible or in the Talmud, proofs for the existence of God are a major topic in the writings of prominent medieval Jewish philosophers. It is instructive to analyze these arguments and their shortcomings. Consider the most famous proof of all – the “prime mover argument.”

We all experience in our daily lives the truism asserted by Aristotle: “There is no motion without a mover.” When I rearrange the living-room furniture under the watchful eye of my wife, I am painfully aware of the fact that the couch will not budge even one centimeter unless I push it, and the instant that I stop pushing, the couch ceases its motion. If I throw a ball, its motion persists momentarily even after it leaves my hand because I have imparted some “impetus” to the ball. According to the widely accepted “impetus theory,” the ball will continue to move until it uses up all its acquired impetus. Then, the ball will come to rest because “there is no motion without a mover.”

Let us now turn our attention to the heavenly bodies, whose ceaseless motion can be observed day after day, year after year, century after century. What causes the ceaseless motion of the heavenly bodies? It must surely be a supernatural entity (God to the medieval theologian; Intelligent Designer in today’s terminology).

The bubble burst in the seventeenth century, when Isaac Newton formulated his famous three laws of motion in the *Principia*, the most important book of science ever written. Newton’s law of inertia states, in contrast to Aristotle, that a moving body *will continue to move forever* unless some force causes it to stop. In the above examples, the force that causes the furniture or the ball to stop moving is friction. However, if friction were not present, then the motion would persist forever. In the heavens, there is no friction. Therefore, according to the law of inertia, heavenly bodies will move forever *without any agency being required to keep them moving*.

To complete the picture, Newton’s law of inertia predicts straight-line motion, whereas the orbit of the planets is an ellipse. This is due to the gravitational attraction between the sun and the planets, which yields the observed elliptical orbits. Planetary motion is completely described by the laws of nature, *without the need to invoke a supernatural entity*. The “prime mover proof” for the existence of God is thus refuted.

## GOD OF THE GAPS

The “prime mover proof” for the existence of God was based on a lack of knowledge of physics. This is an example of what is called the “God of the gaps.” When some phenomenon seems completely inexplicable, one says, “Aha! It must be God Who is causing this phenomenon.” The problem with this approach is that the “completely inexplicable” phenomenon (“gap” in our knowledge) invariably becomes explained as science progresses. As each “gap” in scientific knowledge closes, God is forced to retreat to the next “completely inexplicable” phenomenon. “God of the gaps” arguments thus place God in continual retreat before the relentless advance of science. Surely, this is not the path of a believing person in the search for the Almighty.

This important point is worth emphasizing. Even if one could find no fault in Behe’s claim that IC is completely incompatible with Darwinian evolution, the response of the scientist should be: “Good question! I’ll think about it.” The response should *not* be that of Behe, namely, since I cannot think of a scientific explanation, *it follows* that IC must have been caused by an Intelligent Designer.

## THE JEWISH APPROACH

What is the attitude of leading Jewish scholars today toward possible proofs for the existence of God? Rabbi Joseph B. Soloveitchik^10^ writes that such proofs have never been of any importance to him. As a man of faith, he neither sought nor was he impressed by proofs. Rather, the primary element of faith is to be found within the human spirit. The exhortation “seek and you shall find” is directed *inward*, to the depths of the soul, rather than *outward*, to the logical “proofs” of the philosophers. To Rabbi Soloveitchik, it is the Kierkegaardian “leap of faith” that brings man into communion with the Almighty.

## SCIENCE AND RELIGION

The twelfth-century Jewish theologian and philosopher Moses Maimonides,^11^ after whom this journal is named, wrote that although the believing Jew accepts that Genesis is the word of God, *it does not follow* that he/she must understand every word in Genesis *literally*, because “*the paths of interpretation are not closed to us*.” Maimonides asserted that whenever the literal meaning of the words of Genesis contradicts well-established scientific knowledge, *one should set aside the literal meaning and interpret the Genesis words figuratively.*

Therefore, according to Maimonides, the overwhelming scientific evidence for evolution *does not present any problem at all* to the religious person who believes that the Book of Genesis is the word of God. My own essay^12^ on this subject, entitled “Evolution – Is There a Problem Here?”, ends with this sentence: “It follows that the religious person has no cause to oppose the scientific findings about evolution.”

The reason for the universal opposition to Intelligent Design among scientists is that they view ID as a rejection of science and a return to the ancient world of spirits, deities, and other supernatural beings that were previously proposed to explain many physical phenomena. Scientists look to the laws of nature, and not to supernatural entities, for the explanation of the physical phenomena that they observe.

Jewish tradition confirms the assumption of science that there is regularity to nature and the physical universe operates according to fixed laws (*olam ke’minhago noheg*).^13^ Indeed, Jews are forbidden to depend on a miracle for supplying one’s needs or for solving one’s problems (*ain somchin al ha’nes*).^14^ Praying to God for the occurrence of a supernatural event is denounced in the Talmud as “useless prayer” (*tefilath shav*) and strictly forbidden.^15^

The above paragraph should not be interpreted as implying that God does not interact with the physical world. This is certainly not the case, as Maimonides has stressed. Otherwise, our prayers to God would have no meaning. Thus, the key question is not *whether*, but *how* God influences events.

The Talmud relates to this question by saying that divine providence is bestowed in a manner that is “hidden from the eye” (*samooe min ha’ayin*).^16^ In other words, the framework in which God interacts with the physical world is *within* the laws of nature. Divine intervention rarely involves overtly supernatural events.

Does science assume that miracles do not occur? This would be a serious problem for the religious Jew, because Maimonides^17^ wrote that one who does not believe in the occurrence of miracles is a heretic. How does a religious scientist accommodate science’s assumed regularity of the universe with Maimonides’s dictum about the existence of miracles?

Science does *not* assume that miracles do not occur. Rather, science assumes that the universe *usually* operates through the laws of nature, and one is to ignore entirely the miraculous in seeking explanations for physical phenomena. Thus, my atheist colleague will claim (and that is all that it is – a claim) that miracles never occur, whereas I will claim (based on my religious beliefs) that miracles do occur, at the will of the Almighty, but their occurrence is so rare that miracles do not intrude into my scientific research. The religious scientist never invokes the supernatural as the explanation of any physical phenomenon. He/she recognizes that accepting the existence of miracles is based on religious belief.

Where did the laws of nature come from? Science is silent on this question and *assumes* the existence of laws of nature. The entire enterprise of science is concerned with discovering the laws of nature and with explaining all physical phenomena in terms of these laws.

In fact, there is no *a priori* reason why there should be regularity to nature. Albert Einstein found the existence of laws of nature to be quite surprising, writing: “The most incomprehensible feature of the universe is that it is comprehensible.”^18^

However, the believing person finds deep meaning in the existence of laws of nature and attributes them to God. A well-known religious scientist has written: “The existence of an orderly world, having definite laws of nature, is an expression of the faithfulness of God.”^19^ This statement echoes the words of Genesis 8:22.

Where did the universe come from? Science now has something to say on this question. The universally accepted “big bang” theory of cosmology asserts that the universe had a beginning, which cosmologists commonly refer to as the “creation.”^20^ For example, Nobel laureate Paul Dirac writes: “It seems certain that there was a definite time of creation”.^21^

Science is silent regarding what *caused* the creation. “The creation lies outside the scope of the known laws of physics.”^22^ However, the believing person will see in Dirac’s scientific statement a striking confirmation of the opening verse of Genesis: “*In the beginning, God created the heaven and the earth*.” This opinion of the believer is not related to science, but rather, to faith.

Evolution and cosmology have become established branches of hard science. Judaism has always shown great devotion to science and the pursuit of knowledge. Therefore, Intelligent Design, which denies evolution, has no place in the *weltanschauung* of the religious Jew.

## SUMMARY

Many topics have been covered in this article. It is time to summarize.

1. The proposal of ID has nothing to do with creationism. Neither Behe nor any other proponent of ID ever invoked the words of Genesis as a justification for ID.
2. The proposal of ID has no connection whatsoever with the “argument for design,” except for sharing a common word – design – in its name. The “argument from design” deals with complex systems, *which need not be IC*, whereas ID deals with IC systems, *which need not be complex* (such as the Behe’s simple mouse-trap).
3. The proposal of ID is a “God-of-the-gaps” argument, because Behe invoked the supernatural Intelligent Designer as a result of his inability (gap in his knowledge) to think of a Darwinian explanation for the evolution of an IC system.
4. The religious person who believes that the Book of Genesis is the word of God need not hesitate to accept the scientific findings that demonstrate the evolution of the animal kingdom.
5. The most common proposed refutation of ID, namely, that IC systems are formed by scavenging already existing parts, does not explain most examples of IC (“might happen very, very rarely”).
6. The refutation of ID proposed by H. Allen Orr covers *all cases* of IC, and should therefore be viewed as the definitive refutation. Orr has shown that an IC system can be formed through gradual evolution, with each step offering an additional survival advantage, *even though the final system will not function at all unless every part is present.*

## Abbreviations:

ID: intelligent design;
IC: irreducible complexity
